# The Association Between 5α-Reductase Inhibitors and the Presence of Subsequent Dry Eye Disease in Androgenetic Alopecia: A TriNetX Database Study

**DOI:** 10.7759/cureus.99288

**Published:** 2025-12-15

**Authors:** Chia-Yi Lee, Shun-Fa Yang, Yu-Ling Chang, Jing-Yang Huang, Chao Bin Yeh, Feng-Ming Yeh, Chao Kai Chang

**Affiliations:** 1 Institute of Medicine, Chung Shan Medical University, Taichung, TWN; 2 Department of Pediatric Medicine, Cathay General Hospital, Taipei, TWN; 3 Department of Medical Research, Chung Shan Medical University Hospital, Taichung, TWN; 4 Department of Emergency Medicine, Chung Shan Medical University Hospital, Taichung, TWN; 5 Department of Optometry, Yuanpei University of Medical Technology, Hsinchu, TWN; 6 Department of Ophthalmology, Nobel Eye Institute, Taipei, TWN

**Keywords:** 5α-reductase inhibitors, androgenetic alopecia, dry eye disease, finasteride, trinetx database

## Abstract

Purpose

The purpose of this study is to evaluate the association between the application of 5α-reductase inhibitors (5ARI) and the incidence of subsequent dry eye disease (DED) in the androgenetic alopecia population.

Method

A retrospective cohort study was conducted, and patients with androgenetic alopecia were included and classified according to the receipt of 5ARI therapy or not. A total of 77,982 patients were included in the 5ARI group and the non-5ARI group. The prime outcome was the development of DED after 5ARI usage. The Cox proportional hazard regression was applied for the analysis, which yielded the adjusted hazard ratio (aHR) and 95% confidence interval (CI) of the primary outcome.

Results

After the whole follow-up period up to 10 years, there were 2317 (2.97%) and 2026 (2.60%) DED episodes in the 5ARI group and the non-5ARI group, respectively. The incidence rate is 1.08 per 1000 person-month and 0.84 per 1000 person-month in the 5ARI group and the non-5ARI group, respectively. The 5ARI group demonstrated a higher incidence of DED episode compared to the non-5ARI group after adjustment (aHR: 1.084, 95% CI: 1.039-1.129, P < 0.001). The cumulative probability of the DED development was also significantly higher in the 5ARI group than the non-5ARI group (P < 0.001). In the sensitive analysis, the DED incidences were significantly higher in the 5ARI population than in the non-5ARI population, with several characteristics such as the metabolic syndromes.

Conclusions

The application of 5ARI is associated with a higher risk of following DED development, which is related to the application time in the androgenetic alopecia population.

## Introduction

The hormone accounts for the maintenance of metabolism, tissue remodeling, and reproductive function [1]. Among the hormones of the human body, the androgen is a component that mainly regulates the male characteristics, including the sexual organ development and the development of male secondary sex features [2]. Although the androgen served as an essential hormone, it can contribute to several diseases, such as benign prostate hyperplasia and androgenetic alopecia [3-5]. For the treatment of these diseases, the 5α-reductase inhibitors (5ARI) have been introduced for decades, and the treatment outcome of 5ARI is generally acceptable in previous studies [6,7].

Although the application of 5ARI can reduce the androgen-related diseases, some adverse effects and comorbidities may develop after the 5ARI application [8,9]. In the earlier publication, the usage of 5ARI is associated with a higher incidence of insulin resistance and type 2 diabetes mellitus [9]. Also, the 5ARI application may be correlated to the higher rate of depressive disorder in another study [10]. Besides, a higher incidence of erectile dysfunction and fertility interference was observed in patients who received 5ARI treatment [11]. A recent review article indicates that the 5ARI utilization is related to a tendency of bladder cancer and kidney cancer development [12].

The dry eye disease (DED) is an ocular surface disorder characterized by tear film instability and ocular discomfort [13,14]. In the previous research, the DED could be altered by hormones and hormone replacement therapy [15,16]. Nevertheless, there was a rare study that discussed the relationship between the 5ARI application (like in those with androgenetic alopecia) and the development of DED in clinical practice. Because the 5ARI can regulate the hormones in the human body [17], such an association may exist that needs further evaluation.

Thus, the objective of the current study is to investigate the possible association between the 5ARI application and the incidence of subsequent DED in the androgenetic alopecia patients. Several indicators for the development of DED were also put into the analysis model.

## Materials and methods

Data source

The current study conformed to the Declaration of Helsinki in 1964 and the later amendments. The current study was also accepted by the Institutional Review Board of Chung Shan Medical University (project code: CS2-23180). In this article, C.-Y.L. and S.-F.Y. contribute equally and share the first authorship, and F.-M.Y. and C.-K.C. contribute equally and share the corresponding authorship. The data access is via the specific computer in our institution, and TriNetX governs its reproducibility by providing the same database to all users. The TriNetX database is a global federated health research network allowing access to multiple electronic medical records (diagnoses, laboratory values, procedures, genomic information, medications) throughout major healthcare organizations. The current study was generated by the US Collaborative Network, which contains 108 healthcare institutions. TriNetX is a platform that de-identifies and takes the electronic health document from conducive healthcare systems, most of which are large medical academic institutions with both outpatient and inpatient departments in certain regions in the US. TriNetX Analytics supplies a web-based and secure route to the electronic health records of patients from primary care, hospitals, and specialty treatment providers. Accordingly, the TriNetX database comprises diverse geographical areas, age subgroups, ethnic populations, income degrees, and insurance categories, which involve several commercial insurances, worker governmental insurance (Medicare and Medicaid), compensation insurance, self-pay, uninsured, military affairs insurance, and veterans affairs insurance. The medical data feasible in the TriNetX database include the International Classification of Diseases, Tenth Revision, Clinical Modification (ICD-10-CM) codes, sex, age, employment, residence place, educational degree, socioeconomic and psychosocial circumstances, hospitalization length if recorded, image exam codes, laboratory examination codes, surgery codes, the procedure codes, and the Anatomical Therapeutic Chemical (ATC) codes for medications.

Patient selection

**Figure 1:**
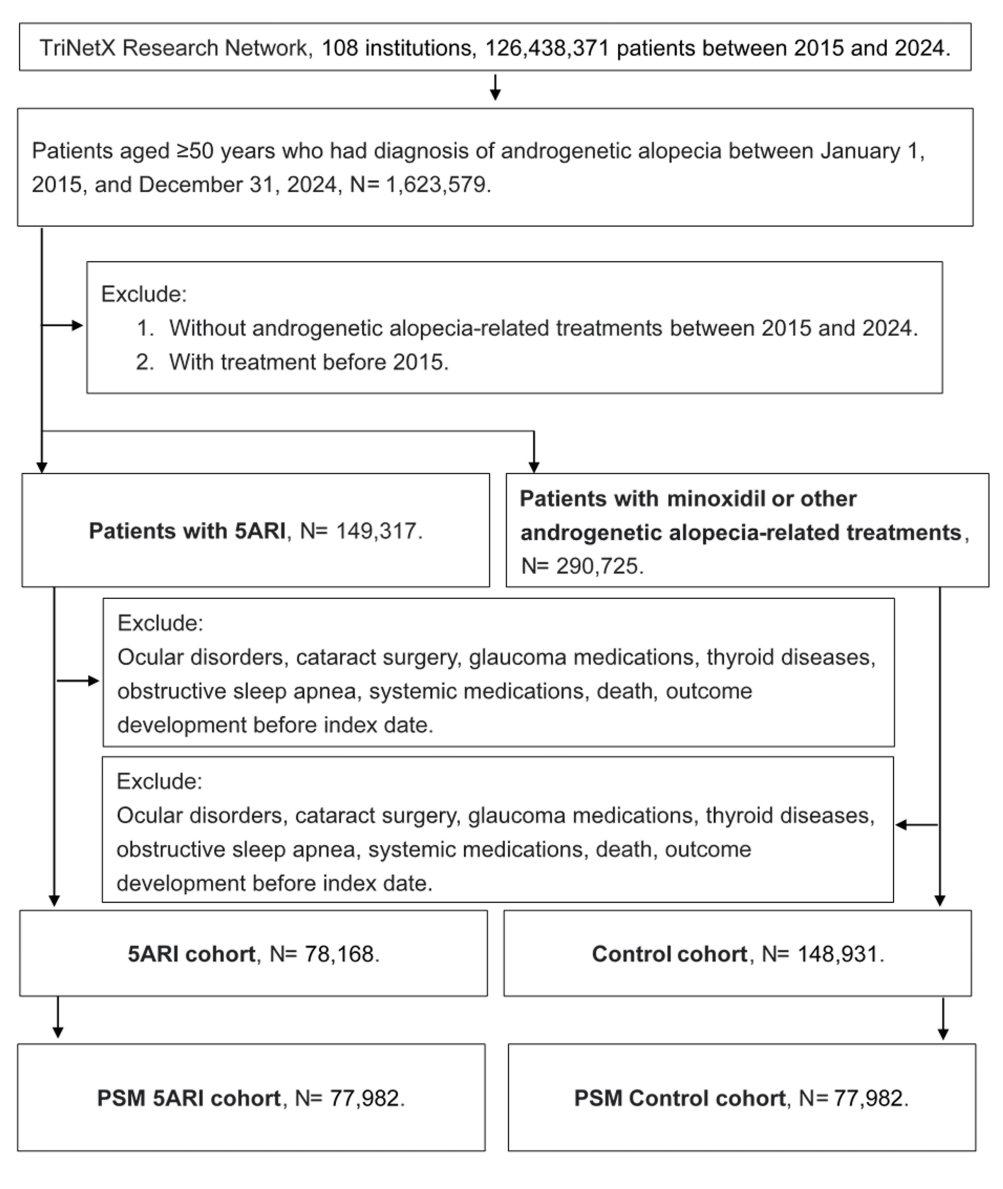
The flowchart of participant selection 5ARI: 5α-reductase inhibitors, N: number, PSM: propensity score matching.

A retrospective cohort study was conducted using the TriNetX database. The patients with the following criteria were selected into the study population: (1) diagnosed with androgenetic alopecia according to the related ICD-10-CM codes, (2) age older than 50 years according to the demographic codes, (3) male sex, and (4) the diagnosis of androgenetic alopecia was made during January 1, 2015, and December 31, 2024. The index date was defined as three months after the diagnosis of androgenetic alopecia. The better standardize the ocular condition of our patients, the following exclusion criteria were applied in the current study: (1) ocular disorders including blindness by any reason, ocular tumor, receipt of eyeball removal surgery (i.e., anophthalmos status), severe ocular trauma, meibomian gland dysfunction, chronic blepharitis, and ocular rosacea before the Index date according to the related ICD-10-CM codes, (2) the receipt of cataract surgery before the index date according to the related surgery codes, (3), the usage of glaucoma medications before the index date according to the related ATC codes, (4) diagnosis of thyroid diseases and obstructive sleep apnea before the index date according to the related ICD-10-CM codes, (5) the usages of systemic medications including the antidepressant medications, antihistamine medications, anticholinergic medications, beta-blockers, and hormonal therapies like (but not limit to) the testosterone replacement before the index date according to the related ATC codes, (5) received treatment before 2015, (6) death before index date according to the related demographic codes, and (7) the outcome (i.e., the DED) or Sjögren syndrome develop before the index date (all the time before the index date, not merely the initial three months after 5ARI usage). The patients were categorized into (1) the 5ARI group, who received the 5ARI treatment according to the ATC codes, and (2) the non-5ARI group, who received the minoxidil or other androgenetic alopecia-related treatments according to the ATC and surgery codes. The 5ARI usage was defined according to the prescriptions recorded, and the patients were required to be new users. We aim to analyze the pharmacological effect of 5ARI; thus, the other patients received therapy other than 5ARI, which comprised the comparison group. After that, the 5ARI group and the non-5ARI group were matched by propensity score-matching (PSM). The PSM cohorts were selected to further balance potential confounders between two study cohorts [18]. We utilized the PROC PSMATCH procedure in SAS software (SAS Institute Inc., Cary, NC) for the propensity score matching The propensity score (probability) of 5ARI exposure was calculated using logistic regression, incorporating covariates such as age at Index date, race, socioeconomic and psychosocial circumstances, nicotine dependence, alcohol related disorders, hypertension, dyslipidemia, diabetes mellitus, ischemic heart diseases, cerebrovascular diseases, unspecific dementia, vascular dementia, rheumatoid arthritis, ankylosing spondylitis and systemic lupus erythematosus. Subsequently, each patient with 5ARI exposure was matched with two non-exposed patients having a similar propensity score, achieved through the nearest neighbor greedy algorithm with a caliper of 0.01. The patient numbers in the 5ARI group and the non-5ARI group were 200,418 and 388,982, respectively, before the PSM process. Finally, a total of 77,982 patients were included in the 5ARI group and the non-5ARI group, respectively. The flowchart of the patient selection is shown in Figure 1.

Prime outcome

The prime outcome of the current study is the development of DED after the index date. The development of DED was defined as the following conditions: (1) the diagnosis of DED according to the related ICD-10-CM codes, (2) the receipt of a tear break-up time exam of Schirmer’s test according to the procedure codes, and (3) the DED diagnosis was made by an ophthalmologist. All the patients were followed until the emergence of the primary outcome, withdrawal from the health care insurance system, or till the end of the TriNetX database, which means December 31, 2024.

Covariates adjustment

To better investigate the association between 5ARI and later DED episodes, the effect of the following covariates was put into the multivariable analysis: age, race, lifestyle factor, socioeconomic and psychosocial circumstances, hypertension, diabetes, ischemic heart disease, hyperlipidemia, cerebrovascular disease, nonspecific dementia, vascular dementia, rheumatoid arthritis, ankylosing spondylitis, and systemic lupus erythematosus. The judgment of the mentioned covariates is according to the related demographic, ICD-10-CM, and the laboratory codes. To ensure the systemic diseases persist long enough to influence the incidence of DED, only the systemic diseases that were recorded for longer than two years were selected for the following analysis.

Statistical analysis

The SAS version 9.4 was utilized in the statistical analyses of the current study. The patient with any missing data would be directly excluded from the current study. The descriptive analysis was utilized to demonstrate the initial features of the 5ARI group and the non-5ARI group, and the standard mean difference (SMD) was utilized to investigate the difference in each feature between the two groups. An SMD higher than 0.1 was set as a significant difference in the current study. Then, the Cox proportional hazard regression was utilized to investigate the incidence of DED development between the 5ARI and non-5ARI groups. The adjusted hazard ratio (aHR) with related 95% confidence interval (CI) for the incidence of DED was then produced. Besides, the age, sex, urbanization, income degree, hypertension, diabetes, ischemic heart disease, hyperlipidemia, cerebrovascular disease, unspecific dementia, vascular dementia, rheumatoid arthritis, ankylosing spondylitis, and systemic lupus erythematosus were selected into the Cox proportional hazard regression to balance their influence on the DED occurrence. The Kaplan-Meier curve was created, and the cumulative incidences of DED episodes between the 5ARI and non-5ARI groups were produced by the log-rank test. In the sensitive analysis, the patients were classified into different subgroups according to age, race, hypertension, diabetes mellitus, dyslipidemia, and autoimmune disease (i.e., rheumatoid arthritis, ankylosing spondylitis, and systemic lupus erythematosus). Then, the Cox proportional hazard regression was utilized again to calculate the risk of DED development in different subgroups. In the current study, deaths, lost to follow-up, and dropout cases were treated as censoring events; when participants died, were lost to follow-up, or withdrew, their data were censored at that time. This standard survival-analysis approach helps reduce bias related to competing risks. The interaction test was applied to analyze the risk of DED between those with metabolic syndromes and those without metabolic syndromes, and among those of different races. Statistical significance was defined as P < 0.05, and the P-value below 0.001 was demonstrated as P < 0.001 in the current study.

## Results

The initial features of the 5ARI group and the non-5ARI group are illustrated in Table 1. The mean age was 70.3 ± 8.2 and 70.8 ± 8.8 years in the 5ARI group and non-5ARI group, respectively. The difference in mean age did not reach statistical significance (SMD = 0.059). The other demographic data, including race, socioeconomic and psychosocial circumstances, and lifestyle factors, also showed non-significant differences between the 5ARI and non-5ARI groups (all SMD < 0.1). About the systemic diseases, the distributions of all diseases were similar between the 5ARI and non-5ARI group (all SMD < 0.1) (Table 1).

**Table 1:** Baseline characteristics among exposure and control cohort after propensity score matching 5α-reductase inhibitors: 5ARI, N: number, SMD: standard mean difference.

Characteristics	5ARI group	Non-5ARI group	SMD
N	77,982	77,982	-
Age at index	70.3 ± 8.2	70.8 ± 8.8	0.059
Race	-	-	-
White	53,767 (68.9%)	53,963 (69.2%)	0.006
Black or African American	6,473 (8.3%)	6,420 (8.2%)	0.004
Asian	6,005 (7.7%)	5,849 (7.5%)	0.004
Socioeconomic and psychosocial circumstances	1,248 (1.6%)	1,080 (1.4%)	0.016
Lifestyle	-	-	-
Nicotine dependence	6,160 (7.9%)	6,003 (7.7%)	0.007
Alcohol related disorders	1,794 (2.3%)	1,708 (2.2%)	0.007
Comorbidities	-	-	-
Hypertension	44,276 (56.8%)	44,200 (56.7%)	0.002
Dyslipidemia	37,561 (48.2%)	37,301 (47.8%)	0.008
Diabetes mellitus	19,724 (25.3%)	19,550 (25.1%)	0.005
Ischemic heart diseases	18,879 (24.2%)	18,697 (24.0%)	0.005
Cerebrovascular diseases	6,706 (8.6%)	6,460 (8.3%)	0.011
Unspecific dementia	2,496 (3.2%)	2,362 (3.0%)	0.012
Vascular dementia	624 (0.8%)	545 (0.7%)	0.012
Rheumatoid arthritis	936 (1.2%)	820 (1.1%)	0.009
Ankylosing spondylitis	156 (0.2%)	142 (0.2%)	0.001
Systemic lupus erythematosus	78 (0.1%)	63 (0.1%)	0.000

**Figure 2:**
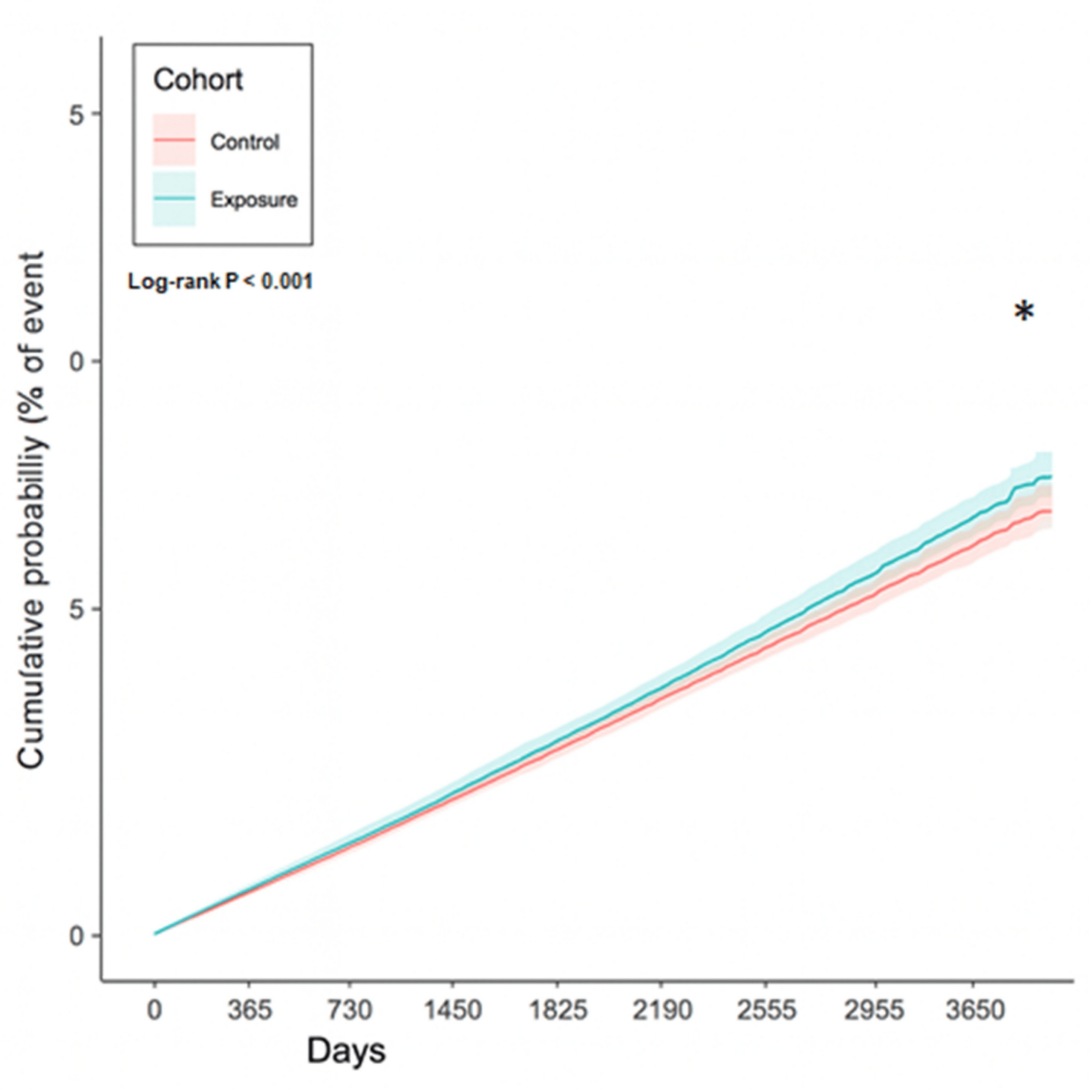
The Kaplan-Meier curve and cumulative incidence of dry eye disease between groups * denotes significant difference between groups.

After the whole follow-up period up to 10 years, there were 2317 (2.97%) and 2026 (2.60%) DED episodes in the 5ARI group and the non-5ARI group, respectively. The incidence rate is 1.08 per 1000 person-month and 0.84 per 1000 person-month in the 5ARI group and the non-5ARI group, respectively. After the adjustment of several covariates, the 5ARI group demonstrated a higher incidence of DED episode compared to the non-5ARI group (aHR: 1.084, 95% CI: 1.039-1.129, P < 0.001) (Table 2). The Kaplan-Meier curve for the DED development between the two groups is demonstrated in Figure 2. The cumulative probability of the DED development was significantly higher in the 5ARI group compared to the non-5ARI group (P < 0.001) (Figure 2).

**Table 2:** Main outcomes between the two groups 5α-reductase inhibitors: 5ARI, aHR: adjusted hazard ratio, CI: confidence interval, N: number. * denotes significant difference between groups. Cox proportional hazard regression was used to calculate the P-values.

Group	N	Cumulative probability			aHR (95% CI)	Test value	P
		One year	Five years	10 years			
5ARI group	2317	1.12 (1.06-1.18)	5.12 (4.95-5.30)	10.30 (9.81-10.79)	1.08 (1.04-1.13)	Z-value	<0.001*
Non-5ARI group	2026	1.01 (0.97-1.05)	4.68 (4.52-4.84)	10.07 (9.52-10.62)	Reference	-	-

**Figure 3:**
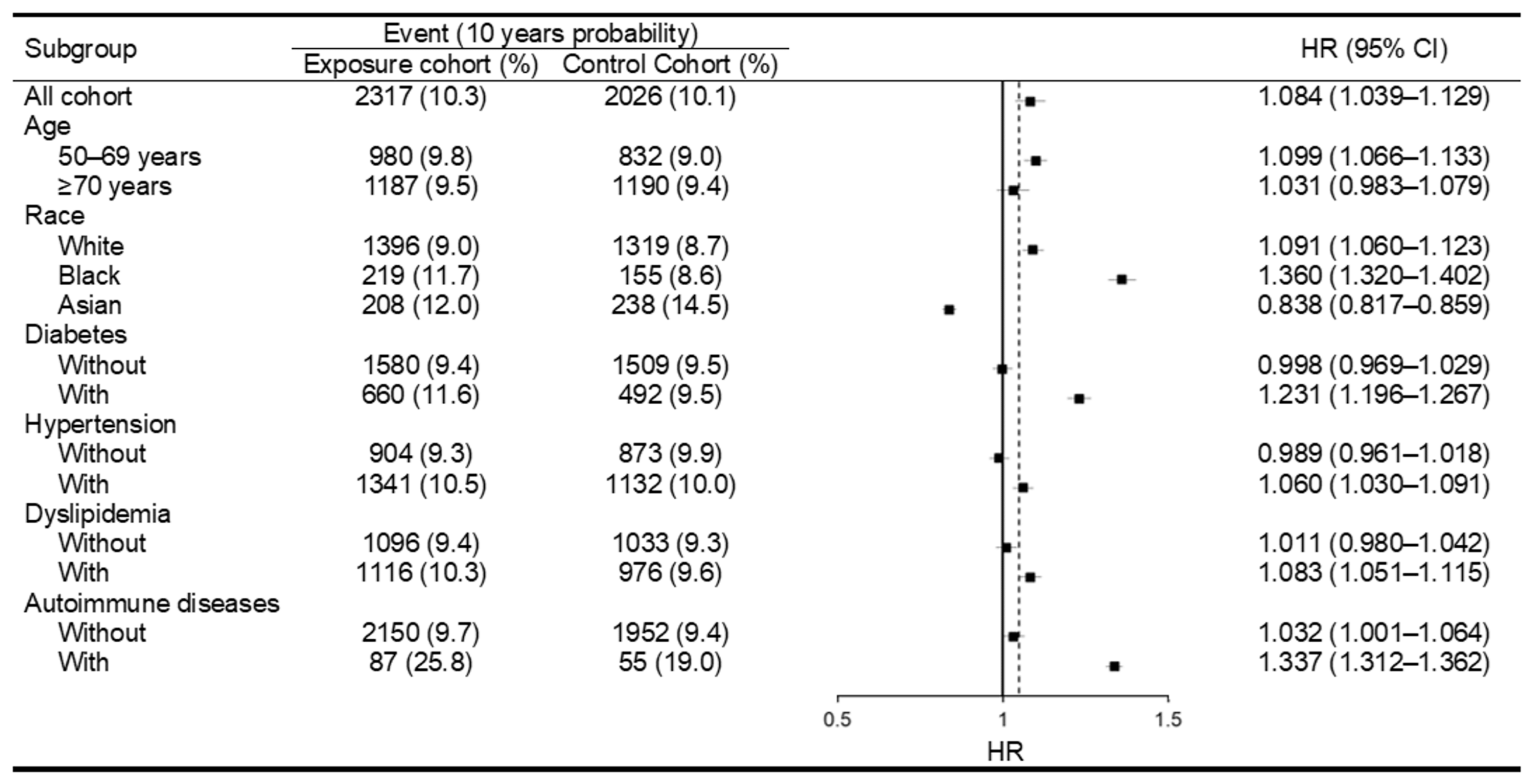
Risk of dry eye disease in androgenetic alopecia patients with 5α-reductase inhibitors usage stratified by age, race, and systemic diseases CI: confidence interval, N: number.

In the sensitive analysis, the DED incidences were significantly higher in the 5ARI population than in the non-5ARI population with different ages and the presence of metabolic diseases (Figure 3). The patients with metabolic syndrome (i.e., hypertension, diabetes mellitus, dyslipidemia) showed a higher risk of DED development than those without metabolic syndrome according to the interaction test (all P < 0.05). On the other side, the risks of developing DED were lower in 5ARI group compared to the non-5ARI groups that own Asian race (aHR: 0.838, 95% CI: 0.817-0.859) (Figure 3), and the interaction test showed a lower incidence of DED with 5ARI usage in Asian population than other two races according to interaction test (P < 0.05).

## Discussion

In the current study, the patients with 5ARI usage demonstrated a higher rate of developing DED compared to those patients without 5ARI application. Moreover, the cumulative probability of DED was significantly higher in the 5ARI group than the non-5ARI group. In addition, the correlation between 5ARI application and DED development was significant in the patients with metabolic syndromes.

The androgen excess is associated with several diseases, whether reproductive organ or not, in the previous publications [5,19]. The benign prostate hyperplasia is featured with androgen excess, urinary inconstancy, and difficulty of urination [19,20]. On the other side, the androgenetic alopecia is mainly featured with androgen excess and hair loss, and the minoxidil and androgen deprivation therapy like finasteride can be applied to treat the androgenetic alopecia [21]. However, the androgen deprivation therapy can also contribute to several complication which including the skin rash, fatigue, muscle weakness and metabolic anomalies [8]. Also, the androgen deprivation therapy application is correlated to the higher possibility of sexual dysfunction [6,8]. In addition, the development of non-alcoholic fatty liver diseases was observed in the patients received androgen deprivation therapy [9]. The DED is an inflammatory ocular disease and the development of DED is reported in several systemic inflammatory diseases like the Sjogren syndrome, rheumatic arthritis, and ankylosing spondylitis [13,22]. For the aspect of hormone, the DED development showed an association to both the estrogen deficiency and androgen deficiency [23-25]. In an experimental study, the application of finasteride, which is a 5ARI that can cause androgen deficiency, contribute to elevated interleukin, elevated tumor necrosis factor-α, and the DED development [26]. This association was also proposed by a later review article [9]. According to the experimental result, we speculate that the usage of 5ARI could correlated to DED development in clinical practice, which is supported by the results of the current study.

In the current study, the application of 5ARI is associated with higher incidence of following DED development. In the previous study evaluating the risk of DED and other medication, the utilization of anti-depressive agent was associated with higher incidence of DED development than the control group [27]. Besides, the elevated sign and symptoms of DED was observed in the individuals that received the hormone replacement therapy in several studies [16]. However, there was rare study to evaluate the correlation between the 5ARI application and the chance of following DED. To our knowledge, our finding may be a preliminary experience to present the positive association between the application of 5ARI and subsequent DED development in patients with androgenetic alopecia, while the absolute risk increase appear small and may be subject to residual or detection bias. Moreover, we adjusted multiple predisposing factor of DED, including the age, sex, diabetes [28,29], in the Cox proportional hazard regression to balance their influence. In addition, only those DED episode that recorded after the index date, thus the time sequence between the 5ARI application and DED development may be confirmed. Consequently, the application of 5ARI may be an independent risk factor for the development of DED. The elevated inflammatory cytokine in those with 5ARI application may be the main reason of the increased DED incidence [26]. Besides, the baseline inflammatory status in the androgenetic alopecia patients were higher than other people [5], thus the dual inflammatory stress of the two diseases plus the 5ARI may enhance the chance of DED development more easily. The cumulative incidence of DED was significantly higher in the 5ARI population than the non-5ARI population. This results may indicate the risk of DED would increase as the duration of 5ARI application persists. Since the application of 5ARI is always long-term, the risk of DED may be high in the population used 5ARI which need attention.

In the sensitive analysis, the risk of DED was significantly higher in the 5ARI population compared to non-5ARI population with different age and the diagnosis of metabolic syndromes according to the interaction test. The age is a known risk factor for the DED development [29], and our result corresponded to the earlier finding. In the previous meta-analysis, the metabolic syndrome, including hypertensive status, dyslipidemia and insulin resistance, is associated with the higher incidence of DED development [30]. The presence of diabetes mellitus was proposed as the risk factor of DED development in another review article [28]. The similar results between our findings and previous experience further support that the metabolic syndromes are an universal risk factor for the DED development in both general population or specific population like patients received 5ARI treatment. On the other side, the Asian population that received 5ARI treatment showed a lower incidence of developing DED than the Asian population that did not received 5ARI treatment, and the Asian 5ARI users demonstrated a lower risk of DED than the 5ARI users with other races. In the previous publications, the Asian ethnicity could be a predisposing factor for the DED development [15,29], and our result is conflicting to previous findings. The possible explanation is that the case numbers of the Asian population in the current study is relatively low. There was near 6,000 Asian men included in both the 5ARI and non-5ARI groups of the current study, which is only account for above 7 percent of the whole study population. The low percentage of Asian men may contribute to statistical bias to some degree. If the percentage of Asian population raised, the statistical result may be different from the result of the current study. Another study that applied Asian people as the main study population is advocated to survey the relationship between the application of 5ARI and DED.

Concerning the epidemiologic prospect, the androgenetic alopecia is occur frequently in the people in which about 80 percent of male in the Western region was suffered from the androgenetic alopecia [6]. Partially due to the androgenetic alopecia, the number of patients that take 5ARI treatment is estimated to be millions in the world [7,21]. On the other side, the DED is a prevalent ocular disease and the overall cases in the world may be tens of millions with and higher incidence in the Asian region [15]. The estimated worldwide prevalence of DED ranged from 5% to 50% [13]. Although the blindness result from DED is not common, the severe DED can contribute to prominent ocular pain, reduced visual acuity and impaired vision-related quality of life [29]. Because the 5ARI treatment was utilized in large population and DED affect the ocular health of the major population significantly, any association between the two conditions should be demonstrated.

There are several limitations in the current study. Firstly, although the statistical results were significant, the aHR was only 1.08 which may indicate that the clinical significance is small. Besides, the current study is a database study which used diagnostic/laboratory/procedure/medication codes as data source rather than real medical document. As a result, multiple important parameters including the medical compliance of patients with androgenetic alopecia, the treatment outcomes of patients with 5ARI treatment, the recurrence of androgenetic alopecia if existed, the details of systemic co-morbidities, the symptoms of DED, the external photography of DED, the results of DED-related examinations, the treatment outcome of DED, the recurrence of DED if existed, contact lens wear if existed, screen time, humidity/airflow exposure, and smoking intensity cannot be evaluated. The detection bias is probably in the current study, as patients receiving 5ARI often have more healthcare contact/visit and therefore greater opportunity for dry eye diagnoses and evaluation. Misclassification bias is also possible since the definition of DED included procedure codes which might be performed for reasons unrelated to DED. Some analyses regarding the dose, duration, categorized cumulative duration, cumulative dose, or time since initiation also cannot be evaluated due to the design of TriNetX database, which could limits interpretation of temporal correlations. Besides, the system of TriNetX can only trace to the time point of DED development, thus the exact treatment that prescribed for the DED patients cannot be taken and analyzed. In addition, the retrospective design of the current study may decrease the homogeneity of our study population significantly and lead to some bias although the baseline characteristics between the two groups were all similar. Finally, we did not perform experimental analysis for the inflammatory cytokines to prove that the higher incidence of DED in 5ARI population is indeed result from inflammation, and the accuracy and integrity of our result and conclusion may be prominently reduced.

## Conclusions

In conclusion, the application of 5ARI is associated with a higher incidence of DED in androgenetic alopecia populations after adjusting for several confounders and lifestyle factors. Furthermore, the risk of DED development in such populations is positively associated with the length of 5ARI treatment, and a higher risk of DED presents in patients who received long-term 5ARI treatment. Consequently, the periodical DED evaluation may be recommended for the androgenetic alopecia patients who received long-term 5ARI treatment to find and properly manage the DED as early as possible. Further large-scale prospective studies to evaluate the influence of 5ARI treatment on the treatment outcome of DED and the additional sensitivity analyses based on stratification (i.e., lag times, alternative outcome definitions, and exclusion of specific populations) for the 5ARI-to-DED relationship in androgenetic alopecia are mandatory.
